# Cognitive behavioural therapy for psychopathology in relatives of missing persons: study protocol for a pilot randomised controlled trial

**DOI:** 10.1186/s40814-016-0055-1

**Published:** 2016-04-01

**Authors:** Lonneke I. M. Lenferink, Ineke Wessel, Jos de Keijser, Paul A. Boelen

**Affiliations:** 1 Department of Clinical Psychology and Experimental Psychopathology, Faculty of Behavioral and Social Sciences, University of Groningen, Grote Kruisstraat 2/1, 9712 TS Groningen, The Netherlands; 2 Department of Clinical Psychology, Faculty of Social Sciences, Utrecht University, P.O. Box 80140, 3508 TC Utrecht, The Netherlands; 3 Arq Psychotrauma Expert Group, Nienoord 5, 1112 XE Diemen, The Netherlands

**Keywords:** Missing persons, Psychopathology, Grief, Cognitive behavioural therapy

## Abstract

**Background:**

It is hypothesized that the grieving process of relatives of missing persons is complicated by having to deal with uncertainty about the fate of their loved one. We developed a cognitive behavioural therapy (CBT) with mindfulness that focuses on dealing with this uncertainty. In this article, we elucidate the rationale of a pilot randomised controlled trial (RCT) for testing the feasibility and potential effectiveness of this CBT for reducing symptoms of psychopathology in relatives of missing persons.

**Methods:**

A pilot RCT comparing participants of the CBT condition (*n* = 15) with waiting list controls (*n* = 15) will be executed. Individuals suffering from psychopathology related to the long-term disappearance of a loved one are eligible to participate. The treatment consists of eight individual sessions. Questionnaires tapping psychological constructs will be administered before, during, and after the treatment. The feasibility of the treatment will be evaluated using descriptive statistics (e.g., attrition rate). The primary analysis consists of a within-group analysis of changes in mean scores of persistent complex bereavement disorder from baseline to immediately post-treatment and follow-up (12 and 24 weeks post-treatment).

**Discussion:**

A significant number of people experience the disappearance of a loved one. Surprisingly, an RCT to evaluate a treatment for psychopathology among relatives of missing persons has never been conducted. Knowledge about treatment effects is needed to improve treatment options for those in need of help. The strengths of this study are the development of a tailored treatment for relatives of missing persons and the use of a pilot design before exposing a large sample to a treatment that has yet to be evaluated. Future research could benefit from the results of this study.

**Trial registration:**

NTR4732 (The Netherlands National Trial Register (NTR))

## Background

The disappearance of a significant other is a potentially devastating loss, due to the lack of knowledge whether the disappeared is dead or alive. Research on the psychological consequences for relatives of the disappeared is scarce and has mainly been conducted in the context of armed conflicts [1–8]. These studies indicate that the disappearance of a significant other is associated with elevated levels of post-traumatic stress disorder (PTSD), major depressive disorder (MDD), and/or disturbed grief.

### Grief following the death or disappearance of a loved one

Whereas little is known about emotional consequences of disappearance, there is a large body of knowledge about both uncomplicated and disturbed grief after the death of a loved one. Grief after the death of a loved one is typically characterized by transient sadness, preoccupation with the circumstances surrounding the loss, and longing for the deceased [9]. People mostly adapt well to the death of a significant other. Nonetheless, about 10 % of the bereaved experience disturbed grief [10, 11]. When grief complaints persist or increase at least 6 months post loss and are associated with distress and impairments in daily functioning, it can be defined as complicated grief (also referred to as prolonged grief disorder) [12]. Although complicated grief partly overlaps with PTSD and MDD, research has shown that these disorders are distinguishable (for overview see [13]). Persistent complex bereavement disorder (PCBD) was included in the fifth edition of the Diagnostic and Statistical Manual of Mental Disorders (DSM-5) as condition for further study [14]. PCBD encompasses persistent separation distress, preoccupation with the loss and the circumstances of the loss beyond 12 months after the loss [14].

There are at least three reasons to assume that grieving the loss of a disappeared relative is more complex and longer lasting than grieving the loss of a deceased relative. To begin with, the on-going uncertainty about the fate of the missing person may lead to preoccupations with his/her potential whereabouts [2, 15]. Constantly thinking about the missing person may exacerbate negative emotions, interfere with daily life tasks and lead to exhaustion [7, 15, 16]. In addition, families of the disappeared are often confronted with financial, emotional, and practical issues for which they receive little professional support [2, 15]. Finally, family conflicts, social marginalization and a lack of social support from the community have been considered to increase the psychological burden [6, 7].

Taken together, compared to individuals bereaved by the death of a loved one, relatives of missing persons might experience more severe PTSD, MDD, and disturbed grief (henceforth referred to as PCBD operationalized as complicated grief). To the best of our knowledge, this hypothesis was tested in only four quantitative studies [3, 5, 6, 17] and was confirmed in two of them [5, 6]. For example, a study among women with unconfirmed and confirmed loss of their husband in a war-related context showed that the former group was more at risk to experience severe MDD symptoms compared to the latter group [5]. In contrast, two other studies did not show significant differences in the severity of symptoms of psychopathology [3, 17]. For example, relatives of Colombians who had disappeared or died in an armed conflict reported similar levels of PCBD, MDD, and PTSD symptoms [3].

There are reasons for doubting that the results of these studies are applicable to relatives of missing persons in general. Firstly, all four studies were conducted in the context of armed conflicts. The participants were highly traumatized by different war-related stressors (e.g., torture). It might therefore be difficult to distinguish the effect of the disappearance from the effects of other traumatic events. Secondly, the studies were conducted in non-Western samples. In general, cultures may differ in how to deal with loss, but more specifically in how to deal with relatives of missing persons [7]. Finally, there is preliminary evidence that relatives who have more hope that their loved one is still alive also experience more severe PCBD complaints [3]. Within the context of war-related disappearances, the majority of the relatives reported being convinced that their loved one was dead [3, 6]. Therefore, relatives who assume that the disappeared person is dead might suffer less from psychological distress than those who assume that the disappeared person might still be alive. Nevertheless, these comparative studies and other studies [2, 4, 7, 8] show that relatives of missing persons might be susceptible for developing symptoms of PCBD, MDD, and PTSD.

One important step would be to design and evaluate an intervention to target these psychopathological symptoms. To the best of our knowledge, this was only done once; a controlled trial compared the effectiveness of two cognitive behavioural therapy (CBT)-based interventions among women who lost a husband due to death or to disappearance during the Srebrenica massacre [18]. Small to medium pre- to post-treatment effect sizes for both conditions were found for disturbed grief and PTSD. However, the methodological drawbacks of this study, including non-random allocation of participants and lack of a threshold of severity of psychopathology as inclusion criterion, need to be taken into account when interpreting the results. Nevertheless, CBT is the treatment of choice for bereavement-related psychopathology [19]. CBT might therefore also be effective in the treatment of psychopathology among relatives of missing persons.

### CBT for relatives of missing persons

A cognitive behavioural theory of PCBD offers a framework for determining variables that should be targeted in CBT among persons who developed PCBD following the death of a significant other [20]. This and several other theories about PCBD highlight the important role of negative cognitions and maladaptive behavioural strategies in the development and persistence of PCBD [20–22]. Cognitive variables include negative views on the self (“I am worthless since he/she died”) and life (“My life has no purpose since he/she died”), a pessimistic view on the future (“I don’t have confidence in the future”), and catastrophic meanings assigned to one’s own reactions to the loss (“If I would elaborate on my feelings, I would lose control”). Maladaptive behavioural strategies include anxious avoidance and depressive avoidance [20]. The former refers to the avoidance of loss-related stimuli out of fear that confrontation with these stimuli will be unbearable. The latter refers to withdrawal from social, recreational, educational, and/or occupational activities fuelled by the belief that these activities are pointless and/or unfulfilling. Problems with integration of the loss into the autobiographical memory are also associated with PCBD. This results in easily triggered intrusive thoughts, images, and memories upon confrontation with loss-related stimuli [20]. Studies among bereaved individuals suffering from PCBD showed the beneficial effect of targeting these cognitive and behavioural variables using CBT [23, 24].

In addition, symptoms of PCBD have been associated with rumination [25, 26]. Rumination encompasses repetitive thinking about one’s negative feelings, their consequences, and/or antecedents [27]. As the disappearance of a person is surrounded by uncertainties, all kinds of repetitive thoughts (e.g., about the whereabouts of the missing person), including ruminative thoughts (e.g., “What am I doing to deserve this?”), might add to the exacerbation and maintenance of symptoms of psychopathology among relatives of missing persons [3, 7, 15, 16]. Rumination can be regarded as a form of “maladaptive coping”, i.e., an unproductive way to master the consequences of the disappearance. Other forms of maladaptive coping that may be pertinent to recovery from the disappearance of a loved one include substance use and suppression of unwanted thoughts and memories.

Although never studied systematically, it is conceivable that these cognitive and behavioural variables are also involved in the maintenance of psychopathology among relatives of missing persons. For instance, relatives of missing persons might no longer perceive the world as a safe place. As a result, they may experience a reduced sense of control and elevated vulnerability. This poses threats to the view of themselves, life, and the future [2, 16]. Relatives may tend to get preoccupied with the missing person and get entangled in repetitive negative thinking and intrusive memories [2, 3, 5, 16]. Consequently, they could withdraw from previously fulfilling activities. Hoping to find the missing person and at the same time coming to terms with the disappearance can result in conflicting feelings [28]. Holding on to hope that the loved one will return might be used as an avoidance strategy to cope with emotions associated with the thought that the separation is permanent [16]. Some avoid discussing what might have happened to the missing loved one, fearing that this may be perceived as giving up hope [29]. Active searching may therefore provide distraction from dwelling on the worst-case scenarios [28].

CBT could give relatives of missing persons insights into how cognitive processes (e.g., thoughts about how they should have prevented the disappearance) affect their emotions (e.g., sadness) and behaviour (e.g., withdrawing from social activities). Unlike treatment for bereavement-related psychopathology, an intervention for relatives of missing persons should not be primarily focused on closure or coming to terms with the irreversibility of the loss. Instead, this treatment ought to be focused on tolerating the ambiguity surrounding the loss and maladaptive repetitive thinking, including thoughts about the whereabouts of the missing person [7, 30]. Adding elements of mindfulness to CBT might serve this treatment aim. In contrast to focusing on external events (e.g., finding out what happened to the missing person), mindfulness is focused on inner psychological experiences (e.g., one’s thoughts, sensations and feelings). Furthermore, mindfulness is not focused on the past or future, but on the present [31]. Mindfulness-based therapy aims to increase the patient’s awareness of his/her inner thoughts, feelings, and bodily sensations in a non-judgemental way through the practice of training in mindfulness meditation [31]. It has frequently shown to reduce levels of psychopathology [32]. A systematic review showed that the beneficial effect of CBT with mindfulness might be, among other variables, due to the reductions of repetitive negative thinking and enhancement of self-compassion [33]. Self-compassion can be viewed as an emotion-regulation strategy [34, 35] that could play a protective role by preventing to become entangled in negative thoughts and emotions [36]. Although these mindfulness-based cognitive therapies have been tested mainly among persons with recurrent depression, preliminary results showed that this approach could also be effective in treatment of grief-related psychopathology [37, 38].

### Study objectives

The aim of this pilot study is to evaluate the feasibility and the potential effectiveness of CBT with elements of mindfulness in reducing PCBD, MDD, and PTSD symptoms and enhancing the extent of mindfulness among relatives of missing persons. Based on previous studies, we expect that the treatment effect will be mediated by changes in negative cognitive and behavioural variables plus enhancement of self-compassion.

The feasibility of the pilot RCT will be explored by the evaluation of the (1) specifics of potential participation bias, (2) attrition rate, (3) methods used and study design, (4) treatment fidelity, and (5) strengths and suggestions for improvements of the treatment from the perspective of the participant. The primary objective of the analysis of the pilot RCT is to assess changes in mean scores on a measure of PCBD from baseline to 1, 12, and 24 weeks post-treatment. A within-group instead of a between-groups analysis will be executed because this will give the most rigorous information about the potential effects of this treatment given the small sample size.

Secondary objectives of the analysis of the pilot RCT are (1) to evaluate whether the mean scores on measures tapping PCBD, MDD, PTSD, and mindfulness of the treatment group differ from the waiting list control group at the post-treatment/post-waiting period assessment when adjusting for the baseline scores and (2) to test whether the treatment effect is mediated by changes in negative cognitive variables (i.e., reductions in negative grief cognitions, intrusive memories, rumination, and repetitive negative thinking) and behavioural variables (i.e., avoidance behaviours) and enhancement of self-compassion. In addition, the extent of change in repetitive negative thinking, intrusive memories, and self-compassion in response to the treatment will be explored at micro-level by means of tracking measures tapping these constructs during the treatment.

## Methods

### Design

This is a multi-centre two-arm pilot RCT exploring the feasibility and potential effectiveness of CBT for relatives of missing persons suffering from psychopathology, in comparison with a waiting list control group. The intervention group will start with the treatment within 1 week after randomisation. The waiting list control group will receive the treatment 12 weeks after randomisation. An allocation ratio of 1:1 will be used. The participants will be asked to fill in questionnaires prior to the treatment and 1, 12, and 24 weeks post-treatment. Participants in the waiting list control group will complete an additional questionnaire 1 week prior to their actual start of the treatment. In addition, the participant will be asked to complete a brief questionnaire at the start of each session to assess the potential change in repetitive negative thinking, intrusive memories, and self-compassion during the treatment.

### Ethics approval

Ethics approval for performing this study has been obtained from the Ethical Committee Psychology at the University of Groningen in the Netherlands (ppo-014-087).

### Participants

First, second, and third degree (adoption- or step-) family members, spouses, and friends of missing persons, who are missing for more than 3 months, are fluent in written and spoken Dutch, and are 18 years of age or older are eligible to sign up for the study. A missing person is defined as “Anyone whose whereabouts is unknown whatever the circumstances of disappearance. They will be considered missing until located and their well-being or otherwise established” (p. 15) [39]. The 3-month criterion was used in a previous study [40] and is also chosen for this study in consultation with representatives of a peer support group and a non-governmental organisation for relatives of missing persons in the Netherlands. Additional inclusion criteria are (1) meeting criteria for PCBD, MDD, and/or PTSD, (2) written informed consent, (3) absence of mental retardation, (4) absence of substance abuse or dependence, (5) absence of psychotic disorder, (6) no high risk of suicide, and (7) no concurrent psychological treatment. In case the missing loved one will be found dead or alive during the period of participation, the participant is offered the opportunity to finish the treatment but will be excluded from further analyses.

### Recruitment of participants

All recruitment procedures aim to enrol individuals of whom a loved one has been missing for participation in a survey study. The survey study aims to explore the psychological consequences for relatives of missing persons. The survey study and pilot RCT are both part of the same research project. We will recruit participants through several pathways. Firstly, representatives from a television show focused on the search of missing persons and a peer support group will distribute invitation letters to relatives of missing persons. Secondly, Victim Support the Netherlands, a governmental organisation in which professionals, mostly social workers, offer practical and legal support to victims, will inform potential participants about the research project. Thirdly, other participants will be recruited via announcements in the media, presentations at meetings for relatives of missing persons, and snowball sampling (each participant is asked to invite others). After signing up for the survey study, the participants will be sent a questionnaire including an information letter and informed consent form for participation in the survey study. The letter informs participants about the aims of the survey study and the possibility to participate in a subsequent study designed to evaluate a psychological intervention specifically developed for relatives of missing persons.

### Procedure and randomisation

After receiving the completed questionnaires and the signed informed consent form for participation in the survey study, the participants will be screened for in- and exclusion criteria for the intervention study. The screening procedure comprises two parts. The first part consists of screening participants for in- and exclusion criteria based on questionnaires (questionnaires and cut-off criteria are described below). Participants who score above the threshold for PCBD, MDD, and/or PTSD will be offered written information about the intervention study together with an informed consent form for participation in the intervention study. The second part of the screening procedure consists of a clinical interview composed of the M.I.N.I. Plus version 5.0.0. and the Traumatic Grief Interview. A trained independent psychologist will conduct the interview by telephone. The M.I.N.I. Plus is developed to diagnose axis I DSM-IV psychiatric disorders and has good psychometric properties [41, 42]. The following modules of the M.I.N.I. Plus will be used: major depressive episode, dysthymia, suicidality, PTSD, alcoholic dependence, substance dependence, and psychotic disorders. In addition, the Traumatic Grief Interview will be administered to assess symptoms of PCBD [43]; based on this interview, participants meet criteria for PCBD when they score a 2 (“sometimes”) or higher on at least 1 B-cluster symptom, at least 6 C-cluster symptoms and a score of 1 (“seldom”) or higher on the D-cluster symptom in accord with the proposed criteria of PCBD in the DSM-5 with exclusion of the criterion that at least 12 months must have been elapsed since the loved one has gone missing [14].

**Fig. 1:**
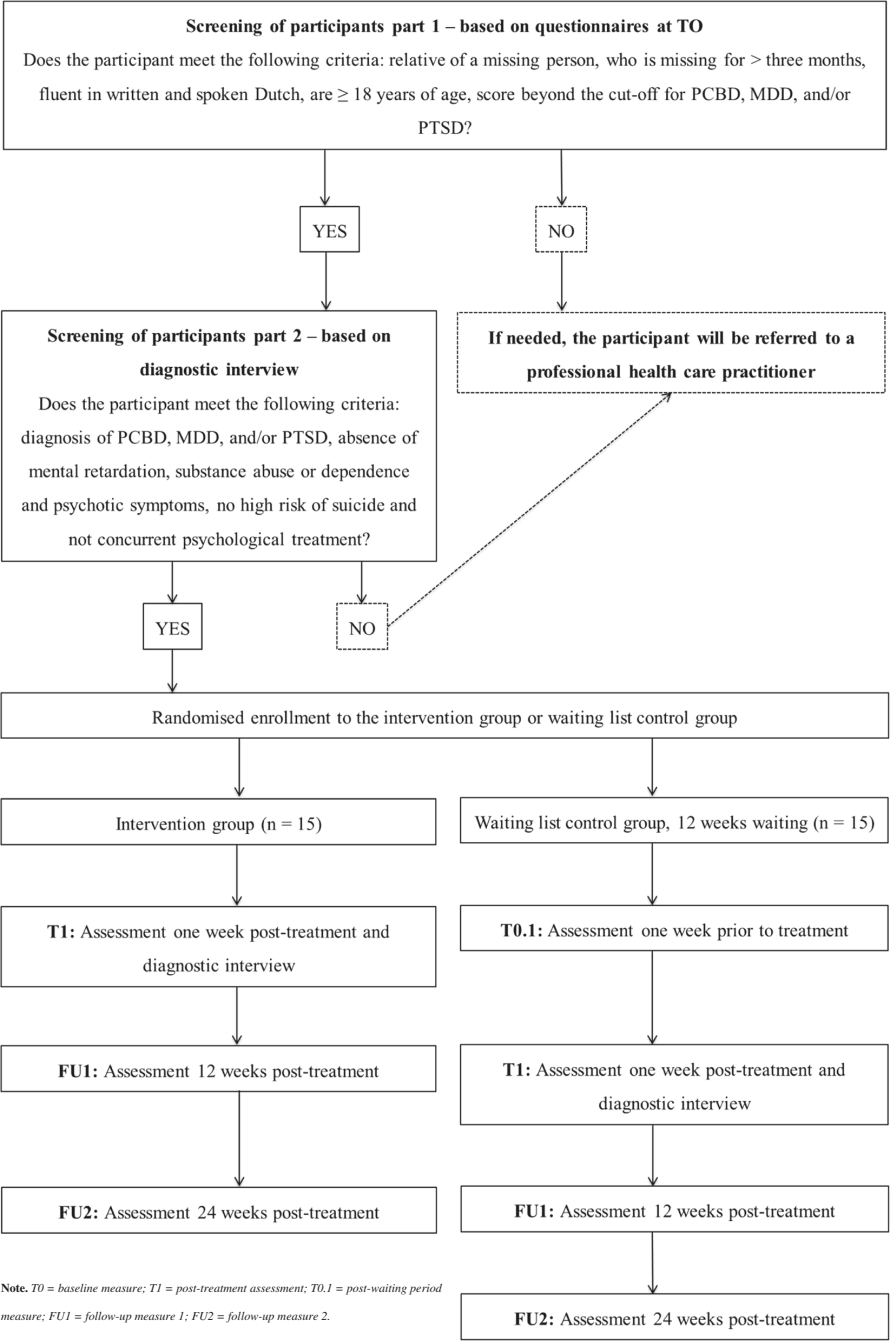
Schematic display of the study procedures

Randomisation will take place after the participant is screened for eligibility based on the M.I.N.I. Plus and the TGI. A random number generator will be used to perform the blocking randomisation procedure. Relatives of the same missing person will be allocated to the same study arm (i.e., intervention or waiting list condition), in order to prevent transfer of information. An independent researcher will conduct the randomisation procedure. Neither the participants nor the researchers will be blinded. The M.I.N.I. Plus and the Traumatic Grief Interview will be conducted again after the treatment, to examine whether numbers of cases of PCBD, MDD, and PTSD have decreased. The researchers will reimburse costs related to the treatment that are not covered by the respondents’ health insurance. Travel expenses that are related to the visits to the therapist will also be reimbursed (see Fig. 1 for a flowchart of the procedures).

### Sample size

To find a within-subjects difference in PCBD symptom across four time points (baseline and 1, 12, and 24 weeks post-treatment) of medium effect size [19] with a power of 80 %, an *α* of 0.05, assuming the correlation between the measures to be 0.50, a sample size of in total 24 participants is sufficient. By taking into account a drop-out rate of 19 %, based on a review that reported a mean attrition rate of 19 % among studies that evaluated the effectiveness of CBT for bereavement-related psychopathology [19], a total sample size of 29 is required.

### Measures

#### Primary outcome measure

##### Inventory of complicated grief

Self-rated symptoms of complicated grief as underlying concept of PCBD will be assessed with the 19-item Inventory of Complicated Grief [44, 45]. Respondents are asked to rate how frequently they experienced 19 grief reactions during the last month, on a 5-point scale ranging from 0 (“never”) to 4 (“always”). The Inventory of Complicated Grief is together with the PG-13 [46] a frequently used instrument to administer grief reactions. Opposed to the former instrument, a validated Dutch translation of the PG-13 is not available. The Dutch translation of the Inventory of Complicated Grief, that demonstrated adequate psychometric properties [45], is therefore chosen as primary outcome measure. We adapted the items of the Inventory of Complicated Grief by referring to the disappearance instead of death (e.g., “Ever since he/she has been missing it is hard for me to trust people”). The Inventory of Complicated Grief is also used as screening instrument in the first part of the screening procedure (see Fig. 1) to assess whether the participant is eligible for the treatment. Participants meet the criteria for PCBD when they score above the cut-off of >25 [44].

#### Secondary outcome measures

##### PTSD Checklist for DSM-5

The severity of PTSD complaints will be assessed with the 20-item PTSD Checklist for DSM-5 [47, 48]. This measure is adapted from the PTSD Checklist for DSM-IV and is in accord with the criteria of PTSD of the DSM-5. Respondents are asked to rate to what extent they experienced PTSD symptoms during the last month on a 5-point scale ranging from 0 (“not at all”) to 4 (“extremely”). A total score (range 0–80) will be obtained by summing item-scores. The initial psychometric properties of the PTSD Checklist for DSM-5 are good [49]. We adapted the wording “the stressful experience” in the instruction and the items to “the events that are associated with the disappearance” (e.g., “In the past month, how much were you bothered by repeated, disturbing, and unwanted memories of the events that are associated with the disappearance?”). The PTSD Checklist for DSM-5 will also be used to screen for eligibility in the first part of the screening procedure (see Fig. 1). The provisional cut-off score of >38 or the diagnostic rule of scoring at least a 2 (“moderately”) on at least 1 cluster B item, 1 cluster C item, 2 cluster D items, and 2 cluster E items will be used as inclusion criteria [48].

##### Inventory of Depressive Symptomatology—Self-report

The severity of depressive symptoms will be assessed with the 30-item Inventory of Depressive Symptomatology—Self-report [50, 51]. Descriptions of depressive symptoms are provided (e.g., *“Feeling sad”*) and respondents are asked to choose an answer that best describes how they felt during the last week (e.g., “I feel sad nearly all of the time”). The items are presented as multiple-choice items, with four options. Total score ranges from 0 to 84 and will be obtained by summing up 28 of the 30 items. This widely used measure has good psychometric properties [51]. The Inventory of Depressive Symptomatology—Self-report will also be used to screen for eligibility in the first part of the screening procedure (see Fig. 1), whereby a score of >13 is defined as an indication of mild depression [52, 53].

##### Southampton Mindfulness Questionnaire

Extent of mindfulness will be assessed with the 16-item Southampton Mindfulness Questionnaire [54, 55]. This instrument is specifically developed to assess changes in the ability to respond mindfully to distressing thoughts and images [54], which are key elements of the treatment (e.g., “Usually when I experience distressing thoughts or images I am able just to notice them without reacting”). Respondents are asked to rate their agreement with each item on a 7-point scale ranging from 0 (“totally agree”) to 6 (“totally disagree”). After reverse coding of some items, a total score (range 0–96) will be obtained by summing the scores for each item. The instrument showed adequate psychometric properties [54].

#### Potential mediators

##### Self-Compassion Scale

The extent of self-compassion will be assessed with the Self-Compassion Scale [56]. The Dutch version of the Self-Compassion Scale consists of 24 items [57] instead of the 26 items in the original version. Respondents are asked to rate how often they behave in the stated manner on a 7-point scale (instead of a 5-point scale in the English version) with anchors “almost never” and “almost always” (e.g., “When I’m feeling down I tend to obsess and fixate on everything that’s wrong”). After reverse coding of some items, the total score (range 24–168) will be obtained by summing all items. The instrument showed adequate psychometric qualities [56].

##### Trauma Memory Questionnaire

The Trauma Memory Questionnaire consists of 13 items divided over two subscales, namely intrusion and disorganization [58, 59]. Only the 8-item intrusion subscale will be administered in this study. We adapted the words that refer to “death” to “disappearance” (e.g., “My memories of the disappearance consist of vivid images”). The items represent different characteristics of intrusive memories associated with the disappearance. Respondents are asked to rate their agreement with each item on a scale ranging from 0 (“not at all”) to 4 (“very strongly”). The original and translated version showed both adequate psychometric properties [58, 59].

##### Grief Cognition Questionnaire

Negative cognitions associated with the disappearance will be assessed with four subscales of the Grief Cognition Questionnaire [60]. We adapted the wording of the items by referring to the disappearance instead of death. The subscales represent negative beliefs about the self (six items, e.g., “Since he/she has been missing, I feel less worthy”), life (four items, e.g., “My life is meaningless since he/she has been missing”), the future (five items, e.g., “I don’t have confidence in the future”) and one’s own grief reactions (four items, e.g., “Once I would start crying, I would lose control”). Respondents rate their agreement with each item on 6-point scales with anchors “disagree strongly” and “agree strongly”. Psychometric properties of this measure are adequate [60].

##### Depressive and Anxious Avoidance in Prolonged Grief Questionnaire

The extent of avoidance behaviour will be assessed with the 9-item Depressive and Anxious Avoidance in Prolonged Grief Questionnaire [61]. As with the other measures, we adapted the words that refer to “death” to “disappearance”. Five items represent depressive avoidance (“I avoid doing activities that used to bring me pleasure, because I feel unable to carry out these activities”) and four items represent anxious avoidance (“I avoid situations and places that confront me with the fact that he/she has been missing and possibly may never return”). Participants rate their agreement with each item on a 6-point scale with anchors “not at all true for me” to “completely true for me”. Psychometric properties of the subscales are adequate [61].

##### Perseverative Thinking Questionnaire

The Perseverative Thinking Questionnaire is a 15-item measure to assess the severity of content-independent repetitive negative thinking [62, 63]. This measure represents the key features of repetitive negative thinking, the perceived unproductiveness of repetitive negative thinking, and the mental capacity that is captured by repetitive negative thinking. Respondents will be asked to rate each item on a 5-point scale ranging from 0 (“never”) to 4 (“almost always”) (e.g., “My thoughts repeat themselves”). The Dutch translation yielded adequate psychometric properties similar to those of the original German and English versions [62, 63].

##### Ruminative Response Scale

The brooding subscale of the Ruminative Response Scale will be administered to measure the tendency to ruminate [64, 65]. Brooding represents a dysfunctional style of depressive rumination [64]. The respondents are instructed to rate what they generally do or think when they feel sad. Respondents will be asked to rate each of the 5 items on a 4-point scale ranging from 1 (“almost never”) to 4 (“almost always”) (e.g., “I think Why do I always react this way?”). Psychometric properties of this measure are adequate [64, 66].

#### Measures used for monitoring treatment progress per session

##### Self-Compassion Scale—Short Form

The 12-item Self-Compassion Scale—Short Form [67] is a shortened version of the Self-Compassion Scale [56]. The Self-Compassion Scale—Short Form consists of two items of each of the six subscales of the extended version. A specific time frame (“the past 7 days”) was added to the instructions in order to monitor the changes in current self-compassion session by session. Psychometric properties of this measure are good [67].

##### Repetitive negative thinking visual analogue scale

We developed the following item to assess the frequency of repetitive negative thinking: “In the past 7 days, to what extent have you been bothered by repeated negative thoughts related to the disappearance of your loved one?”. The item is rated on a visual analogue scale.

##### Characteristics of intrusive memories and images

Six items were developed to assess characteristics of intrusive memories or images during the past 7 days, based on the Intrusive Memory Interview [68]. The first two items assess the content of a recurrent and unwanted memory or image related to the disappearance that was most bothering during the preceding 7 days. First, the participant is asked to write down the content of this memory or image in five key words. Second, the participant is asked to indicate whether the memory or image represents something that “actually”, “possibly”, or “did not happen”. The other four items consist of visual analogue scales that assess the following characteristics of the intrusive memories or images: the extent of distress associated with it, the degree to which they appear to happen in the present, frequency, and vividness (e.g., “How vivid were the intrusions?”).

#### Other measures

Sociodemographic variables (e.g., gender, date of birth, and educational level) of the participants and missing persons will be registered at the first measurement occasion. The number of sessions the participants attended will be registered at the 1-week post-treatment assessment. In case the participant attended less than eight sessions, the reason(s) for drop-out will be registered. The following two open-ended questions regarding the evaluation of the treatment will also be administered at the 1-week post-treatment assessment: “About which aspects of the treatment are you satisfied?” and “About which aspects of the treatment are you less satisfied?”. The questionnaires and time points are summarised in Table 1.

**Table 1:** Overview of variables, concepts, measures, and time points

Variable	Concept	Measure	Time points
Primary outcome measures	Severity of PCBD symptoms	ICG	T0, T0.1, T1, FU1, FU2
Secondary outcome measures	Severity of PTSD symptoms	PCL-5	T0, T0.1, T1, FU1, FU2
	Severity of depressive symptoms	IDS-SR	T0, T0.1, T1, FU1, FU2
	Extent of mindfulness	SMQ	T0, T0.1, T1, FU1, FU2
Mediator	Severity of grief cognitions	GCQ	T0, T0.1, T1, FU1, FU2
	Severity of rumination	RRS	T0, T0.1, T1, FU1, FU2
	Severity of repetitive negative thinking	PTQ	T0, T0.1, T1, FU1, FU2
	Ability to be self-compassionate	SCS	T0, T0.1, T1, FU1, FU2
	Severity of intrusive memories	TMQ	T0, T0.1, T1, FU1, FU2
	Severity of avoidance behaviour	DAAPGQ	T0, T0.1, T1, FU1, FU2
Process of change session by session	Ability to be self-compassionate	SCS-SF	Start of each treatment session
	Severity of intrusive memories and images	Intrusive memories and images vas	Start of each treatment session
	Frequency of repetitive negative thinking	RNT vas	Start of each treatment session
Other variables	Sociodemographic information		T0
	Evaluation of the treatment and reason(s) for drop-out		T1

*ICG* Inventory of Complicated Grief, *PCL-5* Posttraumatic Stress Disorder Checklist for DSM-5, *IDS-SR* Inventory of Depressive Symptomatology—Self-Report, *SMQ* Southampton Mindfulness Questionnaire, *GCQ* Grief Cognition Questionnaire, *RRS* Ruminative Response Scale, *PTQ* Perseverative Thinking Questionnaire, *SCS* Self-Compassion Scale, *TMQ* Trauma Memory Questionnaire, *DAAPGQ* Depressive and Anxious Avoidance in Prolonged Grief Questionnaire, *SCS-SF* Self-Compassion Scale – Short Form, *RNT vas* Repetitive Negative Thinking visual analogue scale, *T0* baseline measure, *T1* post-treatment assessment, *T0.1* post-waiting period measure, *FU1* follow-up measure 1, *FU2* follow-up measure 2

#### Treatment

The treatment is manualized and consists of eight 45-min individual face-to-face therapy sessions offered in a period of maximally 12 weeks. The treatment protocol draws from CBT for treatment of PCBD [23, 69]. Participants learn cognitive behavioural skills and mindfulness practices that enable them to disengage from dysfunctional cognitive patterns related to their missing loved one, both during the sessions and through homework assignments.

In the first session, the therapist and client introduce themselves, exchange expectations regarding the treatment, and the participant is invited to share his/her story about the disappearance of the loved one. The participant receives a manual (containing psycho-education, writing assignments, and exercises for how to handle maladaptive thoughts). Social support is the theme of the second session. The client is asked to invite a relative to join the client in the second session. Mindfulness exercises are introduced in session 3. The therapist explains that mindfulness training consists of sustained attention focused on the body and breath, which integrates a decentred view of thoughts as passing mental events (i.e., “rather than simply being their emotions, or identifying personally with negative thoughts and feelings, patients relate to negative experiences as mental events in a wider context” (p. 276) [70]). A mindfulness exercise is practiced, and the participant will be encouraged to perform mindfulness exercises at home at least five times a week from therapy session 3 to 8. The mindfulness exercises [71] that stem from mindfulness-based cognitive therapy [31] are offered on the website of our research program (www.levenmetvermissing.nl) and via a CD-ROM. The participant is asked to report aspects of their mindfulness-experiences in a mindfulness-diary (e.g., which exercise, duration of the exercise, experiences with the exercise). During session 4 to 8, CBT will be applied that consists of cognitive restructuring and exposure interventions. Cognitive restructuring aims to change dysfunctional thought patterns, and exposure techniques aim to reduce anxiety and avoidance behaviour. Four structured writing assignments are added in a fixed order to the protocol for three reasons. Firstly, the assignments can serve as exposure assignment (e.g., in one of the writing assignments participants are instructed to write about their deepest thoughts and feelings associated with the most stressful memory or image related to the disappearance that keeps popping into their mind) [72]. Secondly, the assignments can increase awareness of inner thoughts and feelings [73, 74]. Lastly, writing about traumatic experiences helps to keep the therapeutic process flowing between the sessions [75]. Two of the writing assignments are imaginary exposure-assignments encouraging participants to tolerate thoughts and feelings related to the disappearance. The first assignment (between session 1 and 2) addresses the impact of the disappearance. The participant is instructed to write about the most intense thoughts and feelings during the first days of the disappearance, followed by a description of how his/her life was before the disappearance and the most painful consequences of the disappearance on his/her life. The second assignment (between session 2 and 3) addresses the description of an image or memory related to the disappearance. The participant is invited to write about the most painful image or memory related to the disappearance and to describe all experiences (thoughts, feelings, and sensations) that come to his/her mind while thinking about that image or memory in as much detail as possible. The third writing assignment (between session 4 and 5) consists of writing a supportive letter to a hypothetical friend that is facing the same situation. This assignment aims to promote the development of new perspectives on the disappearance and its circumstances and to challenge dysfunctional thoughts and behaviour patterns. In the fourth writing assignment (between session 7 and 8), the participant is instructed to write about what he/she has learned during the treatment and how he/she dealt with the disappearance prior to treatment, how he/she deals with it at the moment, and how he/she wishes to deal with it in the future. This final assignment aims to foster empowerment of the participant, in order to deal with possible difficulties in the future. The same sort of writing assignments has been successfully used in treatments of PTSD and PCBD [76, 77].

A group of qualified therapists has received an 8-h training to apply the treatment protocol. The first, third, and last author offered the training. The therapists are all governmentally licensed as psychotherapists or mental healthcare psychologists. They are all part of a network of trained therapists divided over the Netherlands and are experienced in the treatment of PCBD, MDD, and PTSD as a result of extraordinary, high impact loss-related situations.

#### Treatment fidelity

Treatment fidelity will be monitored by asking the therapists to report all compliances and deviations from the treatment protocol in a journal. In addition, supervision takes place every month. Participants are asked to send the writing assignments and mindfulness-diary to the first author at the end of each treatment in order to monitor the compliance.

#### Analyses

##### Feasibility analyses

Analyses regarding the feasibility of this study will primarily be descriptive in terms of attrition rate, reasons for drop-out, and compliance with and deviations from the treatment protocol as well as a summary of the data regarding the strengths and suggestions for improvements of the treatment from the perspective of the participant.

Potential participation bias will be explored by not only comparing relatives who volunteered to participate in the study to those who refused to be in the study with respect to sociodemographic variables, but also by comparing the mean scores of PCBD, MDD, and PTSD.

##### Data analyses

Within-subjects repeated measures ANOVA with time (baseline, 1, 12, and 24 weeks post-treatment) as the repeated measure will be performed to evaluate the effects of the treatment in terms of changes in mean scores of PCBD.

Between-subjects multivariate analysis of covariance (MANCOVA) will be performed to compare changes in PCBD, MDD, PTSDD, and mindfulness scores between the treatment and waiting list condition. Specifically, scores at post-treatment (in the treatment condition) and post-waiting period (in the waiting list condition) on measures tapping these phenomena will be compared, including baseline scores as covariates.

Within-subjects MANCOVA with time (baseline, 1, 12, and 24 weeks post-treatment) as the repeated measure will be performed to evaluate the short- and long-term effects of the treatment in terms of change in mean scores of PCBD, MDD, PTSD, and mindfulness. To test whether the treatment effect is mediated by changes in cognitive variables (i.e., grief cognitions, intrusive memories, rumination, and repetitive negative thinking), behavioural variables (i.e., avoidance behaviour), and self-compassion, only the scores of measures tapping these constructs that show the strongest association (based on univariate analyses) will be added to a multiple regression model. Finally, to assess the potential change at micro-level in repetitive negative thinking, intrusive memories and self-compassion throughout the course of the treatment a within-subjects repeated measures ANOVA with time (session 1 to session 8) as the repeated measure will be performed with mean scores of measures tapping repetitive negative thinking, intrusive memories, and self-compassion as outcome measures.

Data will be analysed according to the intention-to-treat principle. Missing data will be handled using multiple imputations. The Statistical Package for the Social Sciences will be used for the analysis. The small sample size of the pilot RCT increases the risk of type II error; the preliminary results regarding the potential effectiveness will therefore be reported with caution following the recommendations for conducting a pilot study [78].

## Discussion

Worldwide, a significant number of people experience the disappearance of a loved one. Surprisingly, no RCT has ever been conducted to systematically evaluate the effectiveness of a psychological treatment for persistent psychopathology among relatives of missing persons. Knowledge about treatment effects is needed to improve treatment options for those in need of help. This article describes the rationale and methods of a multi-centre pilot RCT focused on the evaluation of the feasibility and preliminary effectiveness of CBT with elements of mindfulness for relatives of missing persons. The treatment is based on CBT for bereavement-related psychopathology and aims to reduce dysfunctional cognitions and avoidance behaviour. Elements of mindfulness are added to the therapy to enhance skills to tolerate the ambiguity surrounding the disappearance and to prevent participants becoming entangled in negative thoughts.

Strengths of this study are (1) the development of a treatment that is targeted to a specific population, (2) the evaluation of the possible mechanisms of change of the treatment, (3) the inclusion of both an intervention and waiting list control group, (4) the use of a pilot design before exposing a large sample to a treatment that has yet to be evaluated, (5) the assessment of eligibility not only by self-report questionnaires but also with a clinical interview, and (6) the monitoring at micro-level of potential change mechanisms of the treatment. One of the limitations of this study is the lack of power to conduct a multilevel analysis to take into account the nested structure of the data (i.e., repeated measures, nested in individuals, who are, in turn, nested in families or other social systems associated with the same missing person). In addition, the recruitment procedures are focused on a non-treatment seeking population. The percentages of individuals who are willing to participate might therefore be relatively low compared to other studies, and the recruitment of sufficient numbers of participants might therefore be a challenge. Moreover, treatment fidelity will be monitored by methods that rely on self-report which might be prone to bias. Finally, participants whose loved one disappeared less than 12 months earlier can formally not fulfil the criteria of PCBD, because one of the proposed criteria of PCBD is that at least 12 months has elapsed since the loss. The reason for this time frame is to distinguish normal from pathological grief [14]. Because little is known about the grief reactions following the disappearance of a loved one, a shorter time frame is chosen to offer support for those in need of help.

As a result of this pilot study, we may have some evidence whether CBT is effective for relatives of missing persons. Since this is a pilot study, the future results of this study need to be interpreted with caution. The recommendations for the execution of a large-scale RCT based on the results of this pilot study are expected to be relevant for future research. Future intervention studies should verify the results of this study and other possibly effective treatments in order to offer an evidence-based intervention for this population. Taken together, this study will provide the first results of a pilot RCT that evaluates the potential effectiveness of CBT for relatives of missing persons suffering from psychopathology.

### Trial status

Recruitment of participants was started in January 2015 and will continue until July 1, 2016.

## Abbreviations

CBT: cognitive behavioural therapy
DSM-5: Fifth Edition of the Diagnostic and Statistical Manual of Mental Disorders
MDD: major depressive disorder
PCBD: persistent complex bereavement disorder
PTSD: post-traumatic stress disorder
RCT: randomised controlled trial
